# Interacting Effects of Cell Size and Temperature on Gene Expression, Growth, Development and Swimming Performance in Larval Zebrafish

**DOI:** 10.3389/fphys.2021.738804

**Published:** 2021-12-07

**Authors:** Iris Louise Eleonora van de Pol, Adam Hermaniuk, Wilhelmus Cornelis Egbertus Petrus Verberk

**Affiliations:** ^1^Department of Animal Ecology and Physiology, Radboud Institute for Biological and Environmental Sciences, Radboud University, Nijmegen, Netherlands; ^2^Department of Evolutionary and Physiological Ecology, Faculty of Biology, University of Białystok, Białystok, Poland

**Keywords:** triploidy, genome size, flow cytometry, thermal biology, ectotherm

## Abstract

Cell size may be important in understanding the thermal biology of ectotherms, as the regulation and consequences of cell size appear to be temperature dependent. Using a recently developed model system of triploid zebrafish (which have around 1.5-fold larger cells than their diploid counterparts) we examine the effects of cell size on gene expression, growth, development and swimming performance in zebrafish larvae at different temperatures. Both temperature and ploidy affected the expression of genes related to metabolic processes (*citrate synthase* and *lactate dehydrogenase*), growth and swimming performance. Temperature also increased development rate, but there was no effect of ploidy level. We did find interactive effects between ploidy and temperature for gene expression, body size and swimming performance, confirming that the consequences of cell size are temperature dependent. Triploids with larger cells performed best at cool conditions, while diploids performed better at warmer conditions. These results suggest different selection pressures on ectotherms and their cell size in cold and warm habitats.

## Introduction

Cell size is emerging as an important trait in understanding the thermal biology of ectothermic animals, as the regulation and consequences of cell size appear to be dependent on temperature (Dufresne and Jeffery, 2011; Hessen et al., 2013; Alfsnes et al., 2017). The predominant factor governing cell size is genome size as the cytoplasmic to nuclear ratio appears to be relatively constant (Gregory, 2001a). Among different animal groups, a large variation in genome sizes is found (Horner and Macgregor, 1983; Gregory, 2001b; Jalal et al., 2015) and in extant fish, the variation in genome size spans the entire breadth of vertebrate genome sizes (Hardie and Hebert, 2004). This large variation in genome size can be partially attributed to whole-genome duplication (WGD) events in the teleost lineage after the divergence of tetrapods (Amores et al., 1998) and polyploidization events in chondrichtheans (cartilaginous fishes) (Stingo and Rocco, 2001), chondrosteans (sturgeons and bichirs) (Blacklidge and Bidwell, 1993) and in certain actinopterygians (ray-finned fishes) (Uyeno and Smith, 1972). However, genome size reductions have also taken place following WGDs as genome size did not exponentially increase DNA content after consecutive WGDs, indicating that genome size and hence cell size is under selection.

Evidence from field studies comparing animals (and plants) with a larger genome and especially polyploids suggests that organisms with a larger genome perform better in colder environments (Bennett, 1976; Gregory and Hebert, 1999): Polyploid amphibians are more abundant at high altitudes or latitudes (Otto et al., 2007), and polyploid *Daphnia pulex* mature faster than their diploid conspecifics, but only at low temperatures (Dufresne and Hebert, 2016). Fish species living in deep, cold water also possess larger cells and genomes than those living in warmer waters (Hardie and Hebert, 2003; Ebeling et al., 2015). Laboratory studies comparing performance of fish with different ploidy levels and cell sizes also indicate that fish with larger cells tend to favor lower temperatures (Atkins and Benfey, 2008; Sambraus et al., 2017). In addition, ectotherms often grow to a larger size in cold environments and this pattern may be especially prominent for aquatic animals with a larger cell size (Verberk et al., 2021). As cell size may be an important determinant for body size, advancing our understanding of cell size adaptations will contribute to understanding the thermal biology of ectotherms.

A role for oxygen in shaping the thermal response in growth is likely (Forster et al., 2012; Horne et al., 2015; Hoefnagel and Verberk, 2015). Oxygen is more likely to become limiting in water than in air, because of the lower diffusion rates in water and its higher viscosity (Verberk et al., 2011). At high temperatures, the metabolic rate of ectotherms increases, which can eventually cause the oxygen demand to exceed supply, which may impair performance (i.e., reduced growth, heat tolerance, fecundity, feeding) (Pörtner, 2010; Verberk et al., 2011). To a certain extent, ectotherms such as fish are able to remodel their physiology to increase oxygen supply, for example by enlarging their gill surface or by increasing hematocrit or ventilating rates (Nilsson, 2007).

Cell size may also affect oxygen supply, as oxygen diffuses more easily across lipid membranes than through aqueous cytosol (Subczynski et al., 1989). Therefore, animals composed of smaller cells may be less susceptible to oxygen limitation, as their membrane surface area is greater than in similarly sized animals composed of large cells. The capacity of individual cells to take up oxygen may be especially relevant in fish larvae that do not have fully developed gills yet and rely heavily on cutaneous oxygen uptake (Rombough, 2002). On the other hand, the larger membrane-to-surface volume ratio in smaller cells makes them more energetically costly to maintain, due to higher costs for lipid turnover and the maintenance of electrochemical gradients across the cell membranes (Szarski, 1983; Hulbert and Else, 2000). Indeed, smaller cells exhibit higher resting metabolic rates on a per mass basis than larger cells (Kozłowski et al., 2003). A previous study on zebrafish larvae with different cell sizes and exposed to different rearing and testing temperatures in a full factorial design found that being composed of larger cells could provide metabolic advantages in the cold, whereas smaller cells are metabolically more beneficial in warm water (Hermaniuk et al., 2021).

This study examined the effects of cell size on gene expression, growth, development and swimming performance in zebrafish larvae reared at different temperatures. We used a triploid zebrafish model that we developed in a previous study (van de Pol et al., 2020). We demonstrated a 1.64 (+0.18 SD) fold increase in erythrocyte cell volume and a 1.72 (+0.70 SD) fold decrease in total nuclei number when comparing triploid larvae to similar sized diploid larvae. Thus, these differences between diploids and triploids closely agree with a 1.5-fold increase in cell size and a 1.5-fold reduction in cell number, that would be expected under a constant nuclear-cytoplasmic ratio. This general pattern of triploid zebrafish larvae having larger but fewer cells need not necessarily apply to all cell types, as we only measured erythrocyte volume directly, but increases in cell volume in polyploid fish have been shown in a range of tissues, including muscle (Suresh and Sheehan, 1998; Vargas et al., 2015), brain and liver (reviewed by Benfey, 1999). Furthermore, triploid zebrafish larvae have been found to be very similar to their diploid counterparts under non-demanding conditions (van de Pol et al., 2020; Small et al., 2021), which makes triploid zebrafish a good model to study the effects of cell size. For this study, we tested whether the consequences of cell size are temperature dependent by rearing and testing the zebrafish at different temperatures. We hypothesize that cell size affects oxygen uptake and transport capacity and therefore triploids with larger cells have a lower energy budget than diploids with smaller cells, especially under conditions where oxygen demand is high (i.e., high temperatures), but not when oxygen demand is lower (i.e., low temperatures), where their greater efficiency can allow them to outperform diploids. Although cold water holds more oxygen, it is also more viscous and oxygen diffusion rates are slower, resulting in a lower bioavailability of oxygen. In addition, and more importantly, the metabolic rate of larvae is reduced in the cold, which means they require less oxygen. This effect of temperature on oxygen demand outweighs the effect on oxygen supply (Verberk et al., 2011). Thus, in the cold the requirement for small cells with high capacity for oxygen uptake is absent, and the larger cells are more beneficial in the cold, as they are more energy efficient. We measured gene expression of metabolic genes, which we considered useful proxies to assess differences in energy budget. We also tested our predictions by comparing diploids and triploids in terms of growth, development, and swimming performance at different temperatures.

## Materials and Methods

### Zebrafish Husbandry, Egg Collection and Triploidy Induction

Maintenance of adult zebrafish and triploidy induction have been described in detail in van de Pol et al. (2020). In brief, zebrafish from the AB strain (supplied by ZIRC, ZFIN ID: ZDB-GENO-960809-7) were kept in 4-L tanks at a density of approximately 30 fish per tank, provided with recirculating tap water (temperature 27°C, pH 7.5–8) under a 14 h:10 h light:dark photoperiod.

Eggs were collected by both natural spawning in mass breeding tanks and *in vitro* fertilization. Triploidy was induced by using a cold shock treatment; 3 min after fertilization the collected eggs were immersed in 4°C E2 medium (5 mmol l^–1^ NaCl, 0.17 mol l^–1^ KCl, and 0.33 mmol l^–1^ MgSO_4_) for 20 min. Eggs were fertilized with a pooled sperm solution of 8–12 males to ensure a heterogeneous offspring. In addition, eggs of different females were randomly distributed over the experimental treatments. Different batches of eggs were harvested from multiple rounds of fertilization, alternating between different parental tanks with similar genetic background. We did not divide the egg batches in half to produce diploids and triploids, due to the vulnerability of the eggs right after fertilization. However, we do not expect family effects as we obtained diploids and triploids from similar parental tanks, although not in the same round of fertilization. Multiple batches of eggs were reared for each experiment: seven for genome size and DNA condensation, two for gene expression, four for development rates, and for growth and swimming performance six batches were reared.

All experiments were carried out in accordance with the Dutch Animals Act^1^, the European guidelines for animal experiments (Directive 2010/63/EU)^2^ and institutional regulations.

### Larval Rearing

Within 2 h after fertilization, diploid and cold shocked embryos were divided over three temperature treatments: 23.5°C, 26.5° (control temperature), and 29.5°C. The embryos were kept in 48-wells plates (Greiner Bio-One, Kremsmünster, Austria) with a mesh bottom and placed in a rearing tank containing E3 medium (E2 medium with addition of 10^–5^% methylene blue). During development, these tanks were constantly aerated and maintained in water baths connected to a circulating heating/cooling system (Grant LT ecocool 150) at the aforementioned temperatures ±0.5°C. Dead embryos or larvae were removed from the setup daily. Survival was affected by ploidy level, but not rearing temperature [see also Hermaniuk et al. (2021) who used the same setup]. We measured length for all larvae used in the swimming trials, enabling us to include length as a co-variate in our statistical models on swimming velocity.

To ensure that larvae were in the same developmental stage in physiological time when performing our experiments, larvae at 23.5°C were reared until 6 days post-fertilization (dpf) and larvae at 29.5°C were reared until 4 dpf, at which point they reached the same developmental stage as 26.5°C larvae reared until 5 dpf. At this time, larvae have almost fully resorbed their yolk sack and are able to produce a proper escape response. Throughout the manuscript, when we indicate 5 dpf, we refer to the developmental stage of 5 dpf for larvae reared at the three different temperatures.

### Genome Size Verification

Triploidy induction was verified in cold shocked larvae at 5 dpf using a propidium iodide (PI) staining to quantify the amount of nuclear DNA. This method has been described in detail in van de Pol et al. (2020). Briefly, a cold shocked and a diploid larva were pooled in one sample, where the diploid serves as an internal control. After homogenization in lysis buffer, the suspension was filtered using a 70 μm cell strainer (pluriSelect Life Science, Leipzig, Germany) to obtain single cell nuclei. These nuclei were stained using PI staining buffer and samples were analyzed with a FC500 5-color Flow Cytometer (Beckman Coulter Life Science, Indianapolis, IN, United States). To verify induction of triploidy, ploidy level was measured on the individual larvae used for the swimming performance trials and growth measurements. Larvae that were cold shocked but turned out to be diploids were excluded from our analyses. The larvae that we used for gene expression and development rate measurements were not verified individually. Instead, triploidy induction efficiency was based on the measurements of 20–30 individual larvae from the same batch. These batch efficiencies ranged from 95.8 to 100%.

In addition to triploidy verification, we also compared the PI staining within diploid and triploid larvae reared at different temperatures. Therefore, we also measured samples containing two diploid larvae for each temperature treatment. Lastly, we calculated the G2/G1 ratio of the cells for each condition, which is a measure for the dividing potential of the cells. The R package flowPloidy (Smith et al., 2018) was used to analyze genome size.

### Gene Expression

Diploid and cold shocked larvae of all three temperature treatments were collected for qPCR analysis at 5 dpf. Two or three larvae were pooled in 2 mL Eppendorf tubes and immediately frozen in liquid nitrogen, with at least five replicate samples per treatment. Details about the qPCR preparation can be found in van de Pol et al. (2020). In short, total RNA isolation with TRIzol (Thermo Fisher Scientific, Waltham, MA, United States) was performed according to the manufacturer’s instruction with some minor changes. RNA concentrations were measured by NanoDrop spectrophotometry (ND-1000; Isogen Life Science B.V., De Meern, Netherlands) to obtain equal amounts of RNA. For cDNA synthesis 500 ng total RNA was used, which was diluted 10x in DEPC H_2_O for qPCR.

The expression levels of six housekeeping genes and four genes related to metabolic processes and temperature responses were analyzed: ribosomal protein S11 (*rps11*, previously known as *40S*); actin, beta 1 (*actb1*); eukaryotic translation elongation factor 1 alpha 1, like 1 (*eef1a1| 1*); polymerase (RNA) II (DNA directed) polypeptide D (*polr2d*); ribosomal protein L13a (*rpl13a*) and TATA box binding protein (*tbp)*. These housekeeping genes are frequently used to normalize gene expression of genes of interest, as they are involved in a range of basic cellular processes. The genes of interest are: citrate synthase, mitochondrial (*cs*); L-lactate dehydrogenase A chain (*ldha*); L-lactate dehydrogenase B-A chain (*ldhba*) and heat shock cognate 70-kd protein, tandem duplicate 1 (*hsp70.1*). Primer sequences and the involvement of these genes in cellular and physiological processes can be found in Table 1.

**TABLE 1:** Primer sequences for qPCR.

Gene	Source	Process	Fw primer sequence (5′-3′)	Rv primer sequence (5′-3′)
*rps11*	NM_213377.1	Translation	GCTTCAAAACCCCCAGAGAA	TCAGGACGTTGAACCTCACA
*actb1*	NM_131031.1	Cytoskeleton integrity	CTTGCTCCTTCCACCATGAA	CTGCTTGCTGATCCACATCT
*eef1a1l1*	NM_131263.1	Cell cycle	CTGGAGGCCAGCTCAAACAT	TCAAGAAGAGTAGTACCGCTAGCATTAC
*polr2d*	NM_001002317.2	Transcription	CCAGATTCAGCCGCTTCAAG	CAAACTGGGAATGAGGGCTT
*rpl13a*	NM_212784.1	Translation	TCTGGAGGACTGTAAGAGGTATGC	AGACGCACAATCTTGAGAGCAG
*tbp*	NM_200096.1	Transcription	CTTACCCACCAGCAGTTTAGCAG	CCTTGGCACCTGTGAGTACGACTTTG
*cs*	NM_199598.1	Aerobic metabolism	ACTCCAACCTGGACTGGTCA	ACGTTTCCACCCTCATGGTC
*ldha*	NM_131246.1	Anaerobic metabolism	GAGACATTCCAGCCCATCCT	ACACCAACCACTGTCACCTT
*ldhba*	NM_131247.1	Anaerobic metabolism	AATCGGGATCATGGCCTCAG	ATCTGCAAGTTCCCGAAGCA
*hsp70.1*	NM_001362359.1	Cellular stress response	GACATCGACGCCAACGGG	GCAGAAATCTTCTCTCTCTGC

To normalize the expression values of the housekeeping genes, the relative quantity of the five other housekeeping genes was used as a combined index. For the genes of interest, relative quantities of all six housekeeping genes were used to normalize expression values, to average out possible variation in housekeeping gene expression caused by the temperature treatments.

### Growth and Development

Development of the embryos was followed up until 72 hours post-fertilization (hpf), by determining their developmental stage according to Kimmel et al. (1995) at fixed time points using a Leica MZ FLIII stereomicroscope (Leica Microsystems, Wetzlar, Germany). For all temperature treatments, staging was performed at 6, 24, 30, 48, and 72 hpf in real-time, to be able to observe temperature effects on development. These time points were chosen because of the clearly distinguishable developmental features, namely: embryonic shield, heart beat and early pigmentation, weak circulation, tapering yolk extension and protruding mouth.

At each time point, five diploid and five cold shocked embryos were staged of all temperature treatments. In our final analysis, we only included development of embryos that morphologically appeared to have developed normally at 5 dpf (e.g., straight body axis, no pericardial edema and normally sized head and eyes). Length was measured at the developmental stage of 5 dpf (protruding mouth stage), using pictures of larvae taken with a Leica MZ FLIII stereomicroscope equipped with a Leica DFC450 C camera and the segmented line tool of the ImageJ program^3^.

### Swimming Performance

Maximum swimming velocity of diploid and cold shocked larvae of the three rearing temperatures was assessed at 5 dpf using a DanioVision system (Noldus Information Technology B.V., Wageningen, Netherlands). Details of the procedure can be found in van de Pol et al. (2020). Briefly, larvae were placed individually in a well of a 24-wells plate (Greiner Bio-One, Kremsmünster, Austria), containing 1 mL of E3 medium. They were presented with 10 tap stimuli with an interval of 20 s [a startle protocol, described in van den Bos et al. (2017)]. The measurement started 10 min after transferring the larvae to the setup. Then they were tracked for 10 min in which no startles were presented, followed by the 10 tap stimuli. In total, this measurement takes 23 min. Their maximum velocity during the startle response was used as a readout, as well as the proportion of responders (velocity above 15 mm/s) and non-responders (velocity below 15 mm/s). Larvae that never showed a response to the tap stimulus were excluded from further analyses. The larvae were measured at the same temperature they were reared at (23.5°C, 26.5°C, and 29.5°C). In a previous study we found the most pronounced differences in metabolic rate between diploids and triploids in larvae reared at 29.5°C and tested at 23.5°C (Hermaniuk et al., 2021). Therefore, we also included this fourth temperature combination in our measurements of swimming performance.

### Statistical Analyses

The collected data were analyzed using RStudio version 1.1.383, respecting a significance threshold of α = 0.05. To compare genome sizes and to test for the effect of rearing temperature on DNA condensation for diploid and triploid larvae, we used a general linear model and subsequent ANOVA. Effects of ploidy and rearing temperature and the interaction between these factors on the ratio of cells in the G2 and G1 phase were also tested with ANOVA. Tukey’s *post hoc* test was performed to compare between the different ploidy and temperature groups.

Gene expression data were analyzed for each gene separately, using a general linear model of which the frequency distribution of the residuals was visually checked for normality. We used a subsequent ANOVA to test for the effects of ploidy level and rearing temperature and the interaction thereof. Tukey’s *post hoc* test was used to annotated differences between ploidy levels and temperature groups.

Development rates of diploid and triploid larvae reared at different temperatures were compared using a general linear model, after visually checking the frequency distribution of the residuals as being normally distributed. We tested for the effects of ploidy level, rearing temperature, development time and the interaction of these factors. Within temperature treatments we tested for the effect of ploidy level, again using general linear models and subsequent ANOVA. Length measurements were analyzed using a general linear model to test for the effects of ploidy level, rearing temperature and the interaction of these factors, as the residuals were normally distributed. We used ANOVA and a subsequent Tukey’s *post hoc* test to compare between the different ploidy and temperature groups.

For the swimming performance data, we first calculated the proportion of diploid and triploid responders vs. non-responders for each temperature treatment and stimulus number, as we observed a bimodality in startle responses (Supplementary Figures 1–3). A linear mixed effects model with a negative binomial structure was used to test for the effects of ploidy level, stimulus number and temperature treatment, including trial as a random factor (a trial was a measurement of a 24-wells plate with diploid or triploid larvae reared at a given temperature). We then analyzed maximum swimming velocity, only for those larvae that showed a startle response at a given stimulus, again using a linear mixed effects model in which we also included length as a factor. For both the proportion of responders and the swimming velocity, we found interactions with temperature treatment. We therefore also analyzed the effect of ploidy level for each temperature treatment by creating subsets of the data. For each subset, we then fitted two models, one with and one without ploidy and used likelihood ratio tests to assess whether the model with ploidy was a significant improvement over the model without ploidy. ANOVA tables for all analyses can be found in Supplementary Tables 1–10.

## Results

### DNA Content and Dividing Potential

**FIGURE 1:**
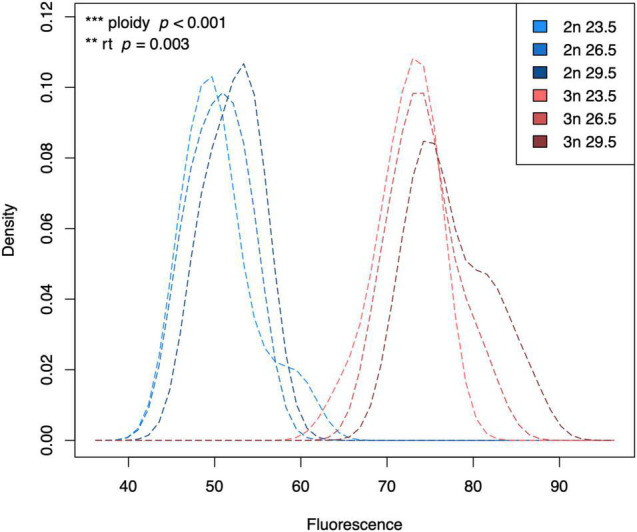
Kernel density plot of the fluorescence of G1 phase cells of diploid and triploid larvae reared at different temperatures. Light, medium, and dark blue lines represent densities for diploid larvae reared at 23.5°C, 26.5°C, and 29.5°C, respectively. Light, medium, and dark red lines represent densities for triploid larvae reared at 23.5°C, 26.5°C, and 29.5°C, respectively. The density is scaled to 1 (100%), which does not have a unit. Fluorescence also does not have a unit, which is why we always add a standard when measuring fluorescence of our samples. Effects of rearing temperature and ploidy were significant (ANOVA, *p* = 0.003 and ANOVA, *p* < 0.001, respectively, *n* = 63).

Flow cytometry clearly distinguished between cells in the G1 phase and cells in the G2 phase (Supplementary Figure 4), with fluorescence intensity mirroring the 1.5 difference in DNA content between diploids and triploids (*F*_1_,_59_ = 650.18, *p* < 0.001, Figure 1). The fluorescence intensity not only reflects genome size, but also the degree of DNA condensation: less propodium iodide can bind to condensed DNA. Focusing on the G1 phase, we found that fluorescence intensity increased with rearing temperature in both diploids and triploids (rearing temperature: *F*_1_,_59_ = 9.57, *p* = 0.003, Figure 1). We did not find an interaction between ploidy and rearing temperature on fluorescence intensity.

**FIGURE 2:**
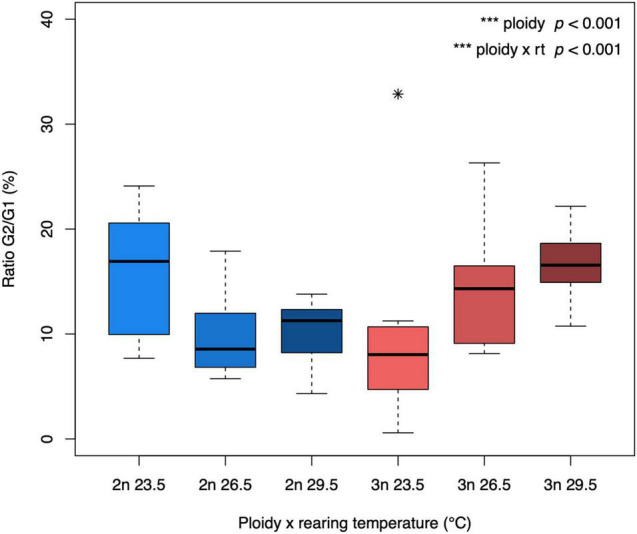
G2/G1 ratio of diploid and triploid larvae reared at different temperatures. The ratio of cells in the G1 and G2 phase was calculated as G2/G1*100 for each larva. The box extends from the lower quartile to the upper quartile of the data, spanning the inter-quartile range (IQR). The thick line within the box represents the median. Whiskers extend to minima and maxima, but are limited to data points 1.5 times outside the IQR. The asterisk represents an outlier. The effect of ploidy and the interaction between ploidy level and rearing temperature were significant (ANOVA, *p* < 0.001, *n* = 64).

The ratio between G2 and G1 phase cells reflects how many cells are in the process of dividing. At the lowest rearing temperature, we found a higher G2/G1 ratio in diploids compared to triploids, but at the highest temperature this pattern was reversed, reflecting a significant interaction between ploidy and rearing temperature (*F*_1_,_58_ = 46.32, *p* < 0.001, Figure 2).

### Expression Levels of Housekeeping Genes and Metabolism Related Genes

Expression levels of the housekeeping genes was used to normalize gene expression of genes of interest (Supplementary Figure 5). Since for some housekeeping genes we also found differences in their expression with rearing temperature and ploidy, we used two normalizations, one based on all housekeeping genes (i.e., *rps11*, *actb1, eef1a1l1, polr2d*, *rpl13a*, and *tbp*; results shown below) and one based on only those genes whose expression did not vary with ploidy and temperature (i.e., *rps11* and *actb1;* results shown in Supplementary Figure 6).

**FIGURE 3:**
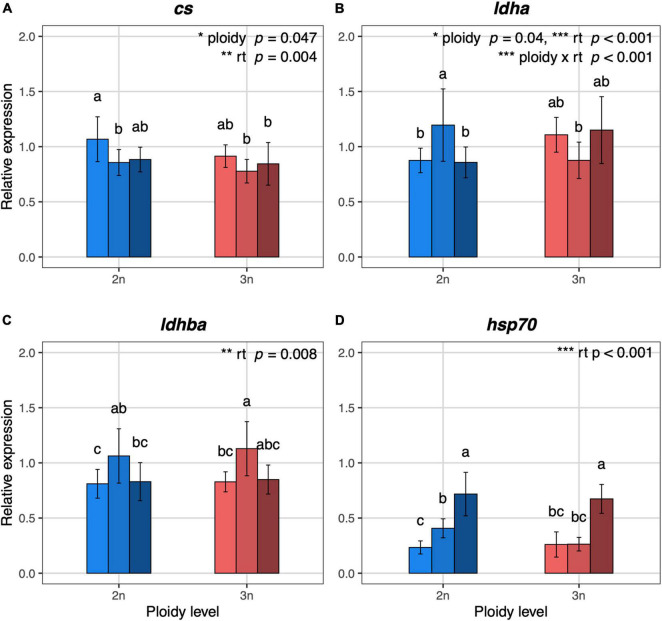
Relative expression values of metabolism and temperature related genes in 5 dpf diploid and triploid larvae reared at different temperatures. **(A)** *cs*, citrate synthase, mitochondrial. **(B)** *ldha*, L-lactate dehydrogenase A chain. **(C)** *ldhba*, L-lactate dehydrogenase B-A chain. **(D)** *hsp70.1*, heat shock cognate 70-kd protein, tandem duplicate 1. Light, medium, and dark blue bars represent expression values for diploid larvae reared at 23.5°C, 26.5°C, and 29.5°C, respectively. Light, medium, and dark red bars represent expression values for triploid larvae reared at 23.5°C, 26.5°C, and 29.5°C, respectively. For each gene, the expression values are normalized using a combined index of the relative quantity of the six housekeeping genes shown in Supplementary Figure 5. Values are represented as means with standard deviations. Rearing temperature and ploidy were significant for *cs* (ANOVA, *p* = 0.004 and *p* = 0.047, respectively, *n* = 48). The interaction between rearing temperature and ploidy, rearing temperature and ploidy were significant for *ldha* (ANOVA, *p* < 0.001, *p* = 0.001, and *p* = 0.04, respectively, *n* = 48). Rearing temperature was significant for *ldhba* and *hsp70.1* (ANOVA, *p* = 0.008 and *p* < 0.001, respectively, *n* = 48; *n* = 47 for hsp70.1). Different letters indicate significant differences between groups (Tukey’s *post hoc* test, *p* < 0.05, *n* = 48).

Expression levels of *cs* were significantly affected by both rearing temperature and ploidy level (*F*_2_,_41_ = 6.11, *p* = 0.004 and *F*_1_,_41_ = 4.19, *p* = 0.047, respectively, Figure 3A), such that the expression of *cs* increased at low temperature in both diploid and triploid larvae. At all temperatures, *cs* expression was lower in triploids. The interaction between temperature and ploidy was not significant. A significant interaction between rearing temperature and ploidy was shown for *ldha* (*F*_2_,_41_ = 9.76, *p* < 0.001, Figure 3B), one of the genes coding for a subunit of the lactate dehygrogenase enzyme, with a reverse pattern for diploids and triploids at the different rearing temperatures. For the expression of *ldhba*, only the effect of rearing temperature was significant (*F*_2_,_41_ = 5.48, *p* = 0.008, Figure 3C), where the lowest expression was found at the higher and lower temperatures. A significant effect of rearing temperature was also found for the expression levels of *hsp70.1* (*F*_2_,_40_ = 41.20, *p* < 0.001, Figure 3D), which increased with temperature.

### Development and Body Length

**FIGURE 4:**
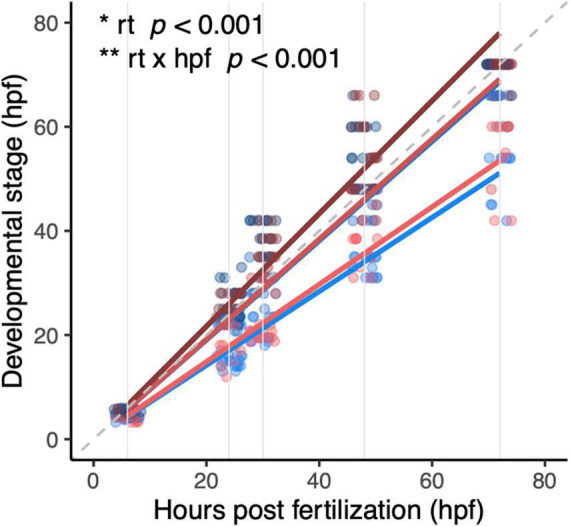
Development of diploid and triploid larvae up to 72 hpf reared at different temperatures. Light, medium, and dark blue lines represent development of diploid larvae reared at 23.5°C, 26.5°C, and 29.5°C, respectively. Light, medium, and dark red lines represent development of triploid larvae reared at 23.5°C, 26.5°C, and 29.5°C, respectively. Note that at 26.5°C and 29.5°C the red and blue lines almost entirely overlap. In this graph, the dashed gray line is the reference x = y. Solid gray lines are the hours post-fertilization at which the embryos and larvae were staged, namely: 6, 24, 30, 48, and 72 h. Effects of rearing temperature and the interaction between hpf and rearing temperature were significant (ANOVA, *p* < 0.001, *n* = 393).

Both diploid and triploid larvae progressed faster through the different developmental stages at higher temperatures (*F*_1_,_386_ = 55.98, *p* < 0.001, Figure 4). There was no significant effect of ploidy (*F*_1_,_386_ = 0.02, *p* = 0.88, Figure 4), and the interaction between rearing temperatures and ploidy level was also not significant (*F*_1_,_386_ = 0.03, *p* = 0.86), indicating that the stimulating effect of warm conditions on development was similar for diploids and triploids.

**FIGURE 5:**
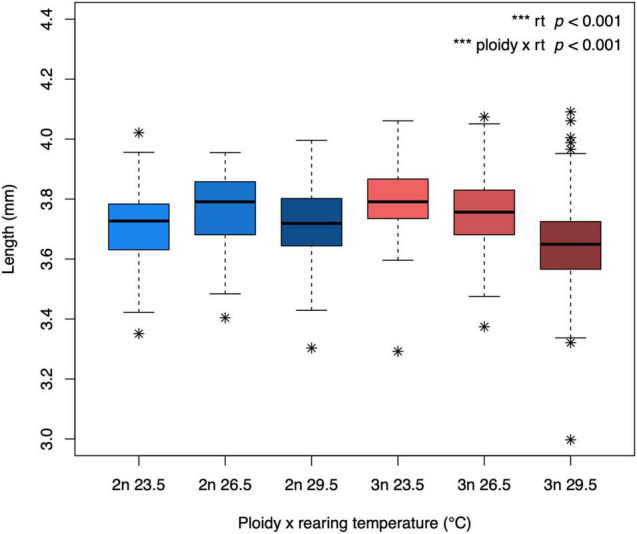
Body length of diploid and triploid larvae reared at different temperatures. Body length was determined at the developmental stage of 5 dpf. The box extends from the lower quartile to the upper quartile of the data, spanning the inter-quartile range (IQR). The thick line within the box represents the median. Whiskers extend to minima and maxima, but are limited to data points 1.5 times outside the IQR. The asterisks represent outliers. The effect of rearing temperature and the interaction between rearing temperature and ploidy level were significant (ANOVA, *p* < 0.001, *n* = 565).

The body length reached at the developmental stage of 5 dpf differed between diploids and triploids, depending on rearing temperature (temperature × ploidy: *F*_1_,_559_ = 23.90, *p* < 0.001, Figure 5). Body length decreased with increasing temperature only in triploids. As a result, triploids tended to reach a larger size in the coldest rearing temperature, but a smaller size in the warmest rearing temperature.

### Swimming Performance

**FIGURE 6:**
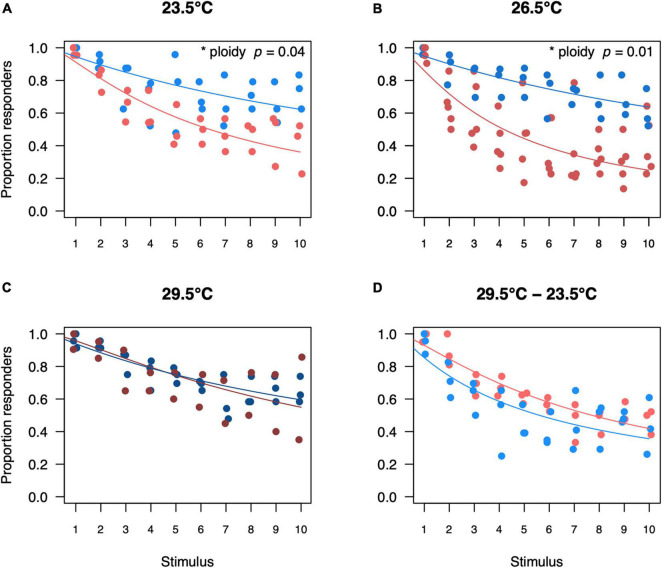
Proportion of diploid and triploid responders for each startle stimulus per temperature treatment. Proportion of responders for diploid and triploid larvae reared and measured at 23.5°C **(A)**, 26.5°C **(B)**, 29.5°C **(C)** or reared at 29.5°C and measured at 23.5°C **(D)**. Depicted values are the proportion of responders per trial, where blue points represent diploid larvae and red points represent triploid larvae. Over all the effect of stimulus number was significant (ANOVA, *p* < 0.001, *n* = 250) as was the interaction between ploidy level and stimulus number (ANOVA, *p* < 0.05, *n* = 250). The effect of ploidy was also analyzed for a subset of each temperature treatment, comparing a model with and without ploidy level included. Ploidy was significant for larvae reared and measured at 23.5°C (ANOVA, *p* = 0.04, *n* = 60) and for larvae reared and measured at 26.5°C (ANOVA, *p* = 0.01, *n* = 80). Ploidy was not significant for larvae reared and measured at 29.5°C (ANOVA, *p* = 0.87, *n* = 50) and for larvae reared at 29.5°C and measured at 23.5°C (ANOVA, *p* = 0.06, *n* = 60).

Upon being startled, most individuals responded by exhibiting an escape response. The proportion of larvae that responded was highest upon the first stimulus and decreased with subsequent stimuli for both ploidy levels and in all temperature treatments (*z* = −13.10, *p* < 0.001, Figure 6). The drop in the proportion of responders was stronger in triploids for the two coolest rearing temperatures, such that diploids were more responsive than triploids (*p* = 0.04 and *p* = 0.01, respectively). This pattern seemed to be reversed at an acute low temperature, although the effect of ploidy was not significant (*p* = 0.06, Figure 6D). All larvae were confirmed to be alive after these trials, as we observed them to swim when transferring them to Eppendorf tubes.

**FIGURE 7:**
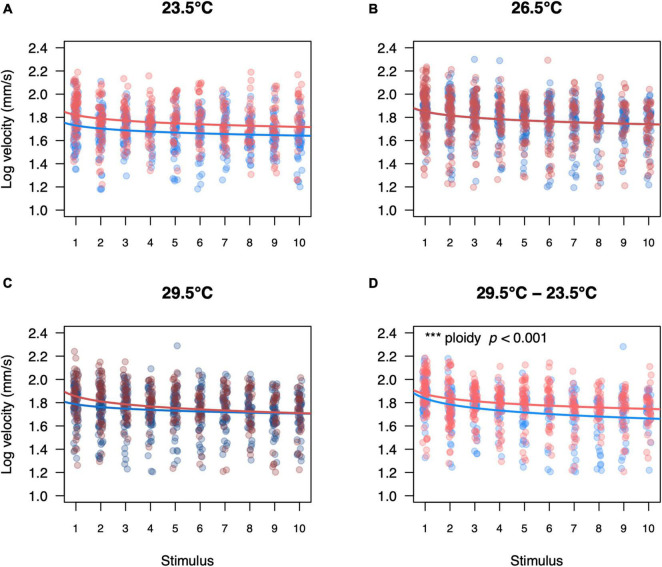
Swimming velocity of diploid and triploid responders for each startle stimulus per temperature treatment. Swimming velocity of diploid and triploid larvae reared and measured at 23.5°C **(A)**, 26.5°C **(B)**, 29.5°C **(C)** or reared at 29.5°C and measured at 23.5°C **(D)**. Each value represents an individual larva that responded to the startle stimulus, where blue points represent diploid larvae and red points represent triploid larvae. Over all the effects of ploidy level (ANOVA, *p* < 0.05), temperature treatment (ANOVA, *p* < 0.05), stimulus number (ANOVA, *p* < 0.001), and length (ANOVA, *p* < 0.001) were significant. The effect of ploidy was also analyzed for a subset of each temperature treatment, comparing a model with and without ploidy level included. Ploidy was significant for larvae reared at 29.5°C and measured at 23.5°C (*p* < 0.001, *n* = 60).

When larvae responded to a stimulus and exhibited an escape response, they could generate an escape velocity of about 63 mm/s (note that the y-axis on Figure 7 are log transformed). Swimming velocity of larvae also decreased with increasing stimuli and this effect was small albeit significant (*F*_1_,_3547_ = 155.96, *p* < 0.001, Figure 7). While the model accounted for potential size differences between triploids and diploids, triploid larvae swam faster when tested at an acute low temperature than diploid larvae (*p* < 0.001, Figure 7D).

## Discussion

Genome size matters for the biology of species. Comparative studies document associations between a species’ genome size and its performance, including a faster development in copepods (Wyngaard et al., 2005), a smaller egg size in fish (Hardie and Hebert, 2003), a higher metabolic rate in amphibians (Gregory, 2003) and a reduced cold tolerance in ectotherms (Leiva et al., 2019). The effects of genome size likely act *via* its correlate cell size, as cell size has consequences for the cellular energy budget: the greater surface area to volume ratio of small cells allows for better cellular uptake of oxygen to fuel aerobic metabolism, but at the same time more energy needs to be expended on maintaining electrochemical gradients. In this study, we tested if the consequences of cell size were dependent on temperature, using triploid zebrafish larvae as a model for zebrafish with larger cells (van de Pol et al., 2020). Our general hypothesis is that having larger cells is advantageous in the cold when energy demand is low, but not in warm conditions that increase energy and hence oxygen demand. Across different levels of biological organization, we indeed found evidence of interactions between rearing temperature and ploidy, confirming the growing idea in the literature that consequences of cell size are temperature dependent.

At the organismal level, we found differences in swimming performance between diploids and triploids. For the first stimulus, almost all larvae exhibited an escape response, but on subsequent stimuli, the proportion of larvae that responded declined, especially in triploids under control conditions at 26.5°C. This suggests that triploid larvae run out of energy sooner. The smaller surface area to volume ratio of their larger cells could limit oxygen transport by erythrocytes and oxygen transport across cell membranes, causing a shortage of oxygen supply to the mitochondria. Although the startle response is a form of burst activity, it is likely fueled predominantly by aerobic metabolism in larval zebrafish. In contrast to adult fish, larval fish have only one layer of red muscle fibers, which show strong cytochrome oxidase activity, instead of a 2-gear muscle system with white and red fibers (El-Fiky et al., 1987; Hunt von Herbing, 2002). In addition, enzyme activity related to glycolysis, indicative of anaerobic metabolism, develops later in ontogeny (El-Fiky et al., 1987; Hinterleitner et al., 1987). The responsiveness after multiple stimuli differed across temperature treatments. In the trials where the larvae were exposed to an acute lower temperature (i.e., reared at 29.5°C, tested at 23.5°C) the triploids were more responsive. Interestingly, at this same temperature combination we previously reported that triploid zebrafish larvae were able to maintain higher metabolic rates than diploids (Hermaniuk et al., 2021). In salmonids (*Salmo salar* and *Salvelinus fontinalis*) triploids are also reported to maintain higher routine metabolic rate at low temperatures compared with diploids (Atkins and Benfey, 2008). Because of their larger cells, triploids should have lower energetic costs for maintaining ionic gradients across cell membranes. The fact that they nevertheless exhibit higher metabolic rates at low temperatures suggests that they have higher energy budgets. Our results seem to confirm this line of thought, as the higher responsiveness of triploids at the acute low measurement temperature indicates that they had more energy available to exhibit a startle response. Moreover, when they exhibited a startle response, the triploids appeared to be slightly faster at the colder test temperatures.

Growth and size attained is another important performance metric (Lefevre et al., 2021; Verberk et al., 2021). Warmer temperatures are known to speed up growth and development such that ectotherms grow faster but reach a smaller size when comparing them at the same stage (Verberk et al., 2021). The effect of temperature on development was taken into account in our experimental design so we could compare larvae reared at different temperatures at the same developmental stage (i.e., 5 dpf at 26.5°C). For a given temperature, there were no clear differences in development rate with ploidy. Similarly, no differences in development rate were reported between developing diploid and triploid tadpoles (*Pelophylax esculentus*) (Hermaniuk et al., 2016). Indeed, developmental processes appear to be remarkably robust at the organismal level when cell sizes are altered (Fankhauser, 1945; Henery et al., 1992; Neufeld et al., 1998). In contrast to development, the effects of temperature on growth and the resultant size did differ with ploidy. Especially triploids attained a larger size at the coldest temperature and vice versa, consistent with a higher energy budget of triploids in the cold (Hermaniuk et al., 2021). A stronger temperature-size response has been previously reported under hypoxia, i.e., conditions were oxygen is more likely to be limiting (Frazier et al., 2001; Hoefnagel and Verberk, 2015). Similarly, comparisons between diploids and triploids have found triploids to be more susceptible to hypoxia (Sambraus et al., 2017) and exhibit stronger temperature-size responses in Hermaniuk et al. (2016). Thus, the stronger temperature-size response in larger celled, aquatic organisms may result from an increased susceptibility to oxygen limitation (Verberk et al., 2021).

Growth and development are ultimately determined by the rate of cell proliferation and cellular differentiation. Given that the timing of development was largely unaffected by ploidy, it follows that size differences likely reflect rates of cell division. In a previous study we found more cells being in the G2 phase in triploids, giving rise to a higher ratio of G2/G1 phases than in diploids (van de Pol et al., 2020). Here we show that this ratio is temperature dependent, and that the relationship with temperature differs between diploids and triploids. In triploids, the G2/G1 ratio was lowest in the cold, while in diploids, the lowest ratios were observed in the two warmest temperatures. We suggest that lower G2/G1 ratios indicate faster growth with less cells being “stuck” in the G2 phase. This would be consistent with the results on size obtained, and suggests that growth was more optimal in cold conditions for triploids and vice versa. Also, for gene expression, we found increased expression levels of the genes *eef1a1| 1* and *rpl13* with higher rearing temperature, both of which are involved in protein synthesis during translation. Although expression levels of genes cannot be directly interpreted as different amounts of protein product from these genes, due to differences in translation and post-translational modifications, the higher expression levels would suggest increased protein synthesis to fuel the faster growth and development at higher temperatures. Interestingly, gene expression of *polr2d*, coding for RNA polymerase II, and *tbp*, coding for TATA box binding protein, were higher at lower temperatures. Possibly, transcription, rather than translation was a rate limiting step in colder temperatures and the higher gene expression helps to maintain transcription and thus enhance cell growth even at lower temperatures (Hessen et al., 2013). There was a tendency for both these genes to be expressed at higher levels in diploids (significant for *tbp*), perhaps compensating for their lower DNA content. Moreover, fluorescence intensity of stained DNA decreased with temperature, suggesting that DNA might be more condensed at lower temperatures. It is possible that the DNA configuration of the larvae at 29.5°C is easier accessible for PI, which is a small molecule, due to more flexible bended DNA (Driessen et al., 2014). Jalal et al. (2015) also reported more condensed DNA in fruit flies reared under low temperature than those raised at high temperature.

Both ploidy and rearing temperature had significant effects on the expression levels of citrate synthase, which is the pacemaker enzyme in the TCA cycle and thus a marker for the aerobic metabolism (Ciccarone et al., 2018). The expression of *cs* increased at low temperature in both diploid and triploid larvae, but this could reflect either a higher activity of the enzyme or constitute a compensatory mechanism to maintain high metabolic rates in cold conditions. For example, McClelland et al. (2006) found that cold-acclimation in adult zebrafish for 4 weeks increased the enzymatic activity of citrate synthase in muscle, but they did not find a significant increase in *cs* expression levels. At all rearing temperatures, *cs* was slightly lower expressed in triploids, which is in line with the lower mass-specific metabolic rate of larger cells (Goniakowska, 1970; Monnickendam and Balls, 1973).

Lactate dehydrogenase is generally used as a marker for anaerobic metabolism, as it is a key enzyme in maintaining cellular homeostasis when oxygen is short in supply by converting pyruvate to lactate. The interaction between rearing temperature and ploidy was significant for *ldha*, the gene coding for the subunit which is predominantly present in muscle and liver tissue (Markert et al., 1975). Taken together, our results could indicate a difference in cellular metabolism and energy fluxes in diploid compared to triploid zebrafish larvae, but the exact mechanisms require further investigation. Finally, expression of *hsp70.1* was significantly elevated at the highest rearing temperature. Although, this increase was similar in triploids and diploids, this suggests that zebrafish already experience thermal stress at 29.5°C, which is quite a lot lower than the critical thermal maximum of 41°C reported by Morgan et al. (2018), but note that these trials were rapid (approx. 45 min) and the upper temperature that an animal can tolerate will be lower at longer timescales (Rezende et al., 2014; Semsar-Kazerouni and Verberk, 2018).

In summary, our study demonstrates that zebrafish larvae with larger cells respond differently to different rearing temperatures than diploids, in terms of their gene expression, growth, development and swimming performance. Likely, this is caused by a different energy budget of their larger cells. A role for oxygen is plausible, as we measured differential expression of citrate synthase, and a previous study demonstrated that oxygen consumption was affected by cell size and temperature in this model system (Hermaniuk et al., 2021). As we studied larvae up until 5 dpf and used relatively short trials to measure swimming performance, some of the subtle differences found in this study may have a larger cumulative effect later in ontogeny. Across all metrics, a general picture emerged, with large celled triploids performing better at low temperatures than small celled diploids. This is consistent with the idea that being composed of larger cells is energetically more efficient at low temperatures. This suggests that the cellular trade-off between high capacity for performance and efficiency is dependent on temperature, and suggest different selection pressures operate on ectotherms and their cell size in cold and warm habitats.

## Data Availability Statement

All data files will be made available in the DANS EASY archive (https://easy.dans.knaw.nl/ui/home, doi: 10.17026/dans-z6g-75da).

## Ethics Statement

Ethical review and approval was not required for the animal study because the experiments were performed with larvae up to a developmental stage of 5 days post-fertilization, a stage where larvae are not yet dependent on external feeding.

## Author Contributions

IP and WV conceived the experimental design and analyzed the data. IP performed the experiments and wrote the first draft of the manuscript. AH participated in collecting data on development rates. WV and AH edited the manuscript. All authors contributed to the article and approved the final version.

## Conflict of Interest

The authors declare that the research was conducted in the absence of any commercial or financial relationships that could be construed as a potential conflict of interest.

## Publisher’s Note

All claims expressed in this article are solely those of the authors and do not necessarily represent those of their affiliated organizations, or those of the publisher, the editors and the reviewers. Any product that may be evaluated in this article, or claim that may be made by its manufacturer, is not guaranteed or endorsed by the publisher.

## Footnotes

1
https://wetten.overheid.nl/BWBR0003081/2019-01-01

2
https://eur-lex.europa.eu/legal-content/EN/TXT/?uri=CELEX:32010L0063

3
https://imagej.nih.gov/ij/
