# Supplementary material for: Interacting Effects of Cell Size and Temperature on Gene Expression, Growth, Development and Swimming Performance in Larval Zebrafish
Source: Front Physiol. 2021 Dec 7;12:738804. doi: 10.3389/fphys.2021.738804 (PMC8691434; doi:10.3389/fphys.2021.738804)
Supplement: Supplementary file 1 [file Data_Sheet_1.docx]

**Supplementary tables**

**Table S1: ANOVA table fluorescence intensity.**

|  | **Sum Sq** | **Df** | **F value** | **Pr(>F)** |
| --- | --- | --- | --- | --- |
| **ploidy** | 8562.82 | 1 | 650.18 | **1.50e-33** |
| **rt** | 126.06 | 1 | 9.57 | **0.003** |
| **ploidy:rt** | 33.13 | 1 | 2.52 | 0.12 |
| **Residuals** | 777.03 | 59 | NA | NA |

**Table S2: ANOVA table G2/G1 ratio.**

|  | **Sum Sq** | **Df** | **F value** | **Pr(>F)** |
| --- | --- | --- | --- | --- |
| **ploidy** | 614.88 | 1 | 45.01 | **8.98e-09** |
| **rt** | 41.90 | 1 | 3.07 | 0.09 |
| **ploidy:rt** | 632.85 | 1 | 46.32 | **7.00e-09** |

**Table S3: ANOVA table expression *cs*.**

|  | **Sum Sq** | **Df** | **F value** | **Pr(>F)** |
| --- | --- | --- | --- | --- |
| **(Intercept)** | 11.40 | 1 | 540.53 | **3.14e-16** |
| **ploidy** | 0.09 | 1 | 4.19 | **0.047** |
| **rt** | 0.26 | 2 | 6.11 | **0.004** |
| **ploidy:rt** | 0.02 | 2 | 0.57 | 0.57 |
| **Residuals** | 0.86 | 41 | NA | NA |

**Table S4: ANOVA table expression *ldha*.**

|  | **Sum Sq** | **Df** | **F value** | **Pr(>F)** |
| --- | --- | --- | --- | --- |
| **(Intercept)** | 7.66 | 1 | 174.32 | **2.35e-16** |
| **ploidy** | 0.20 | 1 | 4.60 | **0.04** |
| **rt** | 0.67 | 2 | 7.65 | **0.001** |
| **ploidy:rt** | 0.86 | 2 | 9.76 | **0.0003** |
| **Residuals** | 1.80 | 41 | NA | NA |

**Table S5: ANOVA table expression *ldhba*.**

|  | **Sum Sq** | **Df** | **F value** | **Pr(>F)** |
| --- | --- | --- | --- | --- |
| **(Intercept)** | 6.57 | 1 | 195.93 | **3.27e-17** |
| **ploidy** | 0.001 | 1 | 0.03 | 0.85 |
| **rt** | 0.37 | 2 | 5.48 | **0.008** |
| **ploidy:rt** | 0.006 | 2 | 0.09 | 0.92 |
| **Residuals** | 1.37 | 41 | NA | NA |

**Table S6: ANOVA table expression *hsp70.1*.**

|  | **Sum Sq** | **Df** | **F value** | **Pr(>F)** |
| --- | --- | --- | --- | --- |
| **(Intercept)** | 0.55 | 1 | 37.42 | **3.24e-07** |
| **ploidy** | 0.002 | 1 | 0.16 | 0.69 |
| **rt** | 1.20 | 2 | 41.20 | **1.93e-10** |
| **ploidy:rt** | 0.05 | 2 | 1.84 | 0.17 |
| **Residuals** | 0.58 | 40 | NA | NA |

**Table S7: ANOVA table development rate.**

|  | **Sum Sq** | **Df** | **F value** | **Pr(>F)** |
| --- | --- | --- | --- | --- |
| **(Intercept)** | 414.90 | 1 | 24.98 | **8.80e-07** |
| **hpf** | 16.95 | 1 | 1.02 | 0.31 |
| **rt** | 440.74 | 1 | 26.54 | **4.13e-07** |
| **ploidy** | 0.40 | 1 | 0.02 | 0.88 |
| **hpf:rt** | 929.63 | 1 | 55.98 | **5.02e-13** |
| **hpf:ploidy** | 6.74 | 1 | 0.41 | 0.52 |
| **rt:ploidy** | 0.52 | 1 | 0.03 | 0.86 |
| **hpf:rt:ploidy** | 4.97 | 1 | 0.30 | 0.58 |
| **Residuals** | 6393.83 | 385 | NA | NA |

**Table S8: ANOVA table length.**

|  | **Sum Sq** | **Df** | **F value** | **Pr(>F)** |
| --- | --- | --- | --- | --- |
| **ploidy** | 0.002 | 1 | 0.09 | 0.76 |
| **rt** | 0.59 | 1 | 33.89 | **9.82e-09** |
| **ploidy:rt** | 0.42 | 1 | 23.90 | **1.33e-06** |
| **Residuals** | 9.84 | 561 | NA | NA |

**Table S9: Summary table responders.**

|  | **Estimate** | **Std. Error** | **z value** | **Pr(>\|z\|)** |
| --- | --- | --- | --- | --- |
| **(Intercept)** | 2.86 | 0.28 | 10.11 | **5.24e-24** |
| **ploidy3n** | -0.52 | 0.39 | -1.33 | 0.18 |
| **logrank** | -1.02 | 0.08 | -13.10 | **3.43e-39** |
| **ctemp26.5 26.5** | 0.06 | 0.35 | 0.16 | 0.87 |
| **ctemp29.5 29.5** | -0.12 | 0.35 | -0.34 | 0.73 |
| **ctemp29.5 23.5** | -1.10 | 0.35 | -3.15 | **0.002** |
| **ploidy3n:logrank** | -0.24 | 0.11 | -2.23 | **0.03** |
| **ploidy3n:ctemp26.5 26.5** | -0.53 | 0.47 | -1.13 | 0.26 |
| **ploidy3n:ctemp29.5 29.5** | 0.77 | 0.52 | 1.46 | 0.14 |
| **ploidy3n:ctemp29.5 23.5** | 1.34 | 0.49 | 2.72 | **0.006** |

**Table S10: ANOVA table speed**

|  | **Sum Sq** | **Df** | **F value** | **Pr(>F)** |
| --- | --- | --- | --- | --- |
| **ploidy** | 0.18 | 1 | 5.74 | **0.02** |
| **ctemp** | 0.31 | 3 | 3.32 | **0.03** |
| **logrank** | 4.90 | 1 | 155.96 | **4.68e-35** |
| **length** | 0.63 | 1 | 20.16 | **7.36e-06** |
| **ctemp:logrank** | 0.15 | 3 | 1.58 | 0.19 |
| **ploidy:ctemp** | 0.09 | 3 | 0.98 | 0.42 |
| **ploidy:logrank** | 0.008 | 1 | 0.24 | 0.62 |
| **ploidy:ctemp:logrank** | 0.13 | 3 | 1.34 | 0.26 |
