# Supplementary material for: Interacting Effects of Cell Size and Temperature on Gene Expression, Growth, Development and Swimming Performance in Larval Zebrafish
Source: Front Physiol. 2021 Dec 7;12:738804. doi: 10.3389/fphys.2021.738804 (PMC8691434; doi:10.3389/fphys.2021.738804)
Supplement: Supplementary file 2 [file Data_Sheet_2.docx]

**Supplementary figures**

**Figure S1-S3:** Bimodal distribution of responders and non-responders.

**Figure S1: Swimming velocity of diploid and triploid larvae at 23.5°C per startle.** Each value represents the velocity of an individual larva following the startle stimulus, where black points represent diploid larvae and red points represent triploid larvae.

**Figure S2: Swimming velocity of diploid and triploid larvae at 26.5°C per startle.** Each value represents the velocity of an individual larva following the startle stimulus, where black points represent diploid larvae and red points represent triploid larvae.

**Figure S3: Swimming velocity of diploid and triploid larvae reared at 29.5°C, measured at 23.5°C per startle.** Each value represents the velocity of an individual larva following the startle stimulus, where black points represent larvae measured at 23.5°C and red points represent larvae measured at 29.5°C.

**Figure S4:** Exemplary flow cytometry output, which allows us to distinguish between diploid and triploid larvae.


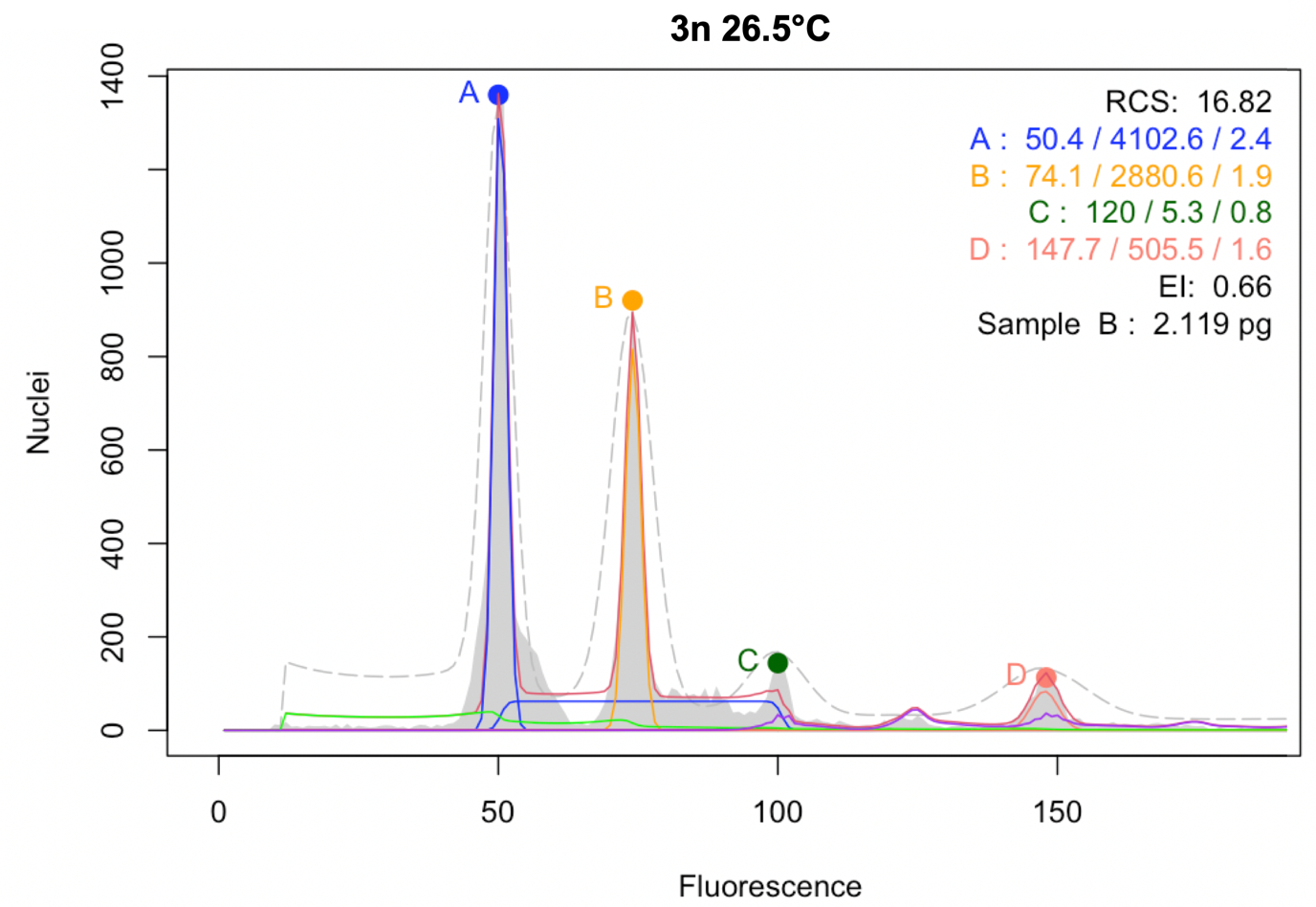


**Figure S4: Exemplary DNA histogram of one diploid and one triploid larva pooled in one sample.** Standard size peak A (G1-peak 2n) = 1.44 pg, estimated size peak B (G1-peak 3n) = 2.12 pg, ratio G1-3n / G1-2n = 1.47. Peak C and Peak D represent G2 phase nuclei of the diploid and triploid larvae, respectively. Fluorescence/cell number/coefficient of variation for each peak are depicted in upper right corner.

**Figure S5 and S6:** Gene expression of housekeeping genes and genes of interest.

**Figure S5: Relative expression values of housekeeping genes in 5 dpf diploid and triploid larvae reared at different temperatures.** A) *rps11*, ribosomal protein S11. B) *actb1*, actin, beta 1. C) *eef1a1|1*, eukaryotic translation elongation factor 1 alpha 1, like 1. D) *polr2d*, polymerase (RNA) II (DNA directed) polypeptide D. E) *rpl13a*, ribosomal protein L13a. F) *tbp*, TATA box binding protein. Blue bars represent diploid larvae, red bars represent triploid larvae. For each gene, the expression values are normalized using a combined index of the relative quantity of the other five housekeeping genes. Values are represented as means with standard deviations. Rearing temperature was significant for *eef1a1|1*, *polr2d* and *rpl13a* (ANOVA, p < 0.05, n = 48). Both ploidy and rearing temperature were significant for *tbp* (ANOVA, p < 0.05, n = 48). Different letters indicate significant differences between groups (Tukey’s post-hoc test, (p < 0.05, n = 48).

Expression levels of *rps11* and *actb1* were similar for 5 dpf diploid and triploid larvae reared at different temperatures (figure S5A and S5B). Rearing temperature had a significant effect on the expression of *eef1a1l1* (F_2,41_ = 18.95, p < 0.001), *polr2d* (F_2,41_ = 15.76, p < 0.001) and *rpl13a* (F_2,41_ = 5.3827, p < 0.01, figure S5C, S5D and S5E), but for these genes no effect of ploidy was found. Both rearing temperature and ploidy had a significant effect on the expression of *tbp* (F_2,41_ = 31.43, p < 0.001 and F_1,41_ = 13.75, p < 0.001, respectively, figure S5F), but the interaction was not significant.

**Figure S6: Relative expression values of metabolism and temperature related genes in 5 dpf diploid and triploid larvae reared at different temperatures, normalized for *rps11* and *actb1*.** A) *cs*, citrate synthase, mitochondrial. B) *ldha*, L-lactate dehydrogenase A chain. C) *ldhba*, L-lactate dehydrogenase B-A chain. D) *hsp70.1*, Heat shock cognate 70-kd protein, tandem duplicate 1. Blue bars represent diploid larvae, red bars represent triploid larvae. For each gene, the expression values are normalized using a combined index of the relative quantity of the six housekeeping genes shown in figure 1. Values are represented as means with standard deviations. Rearing temperature and ploidy were significant for *cs* (ANOVA, *p* < 0.05, n = 48). The interaction between rearing temperature and ploidy was significant for *ldha* (ANOVA, *p* < 0.05, n = 48). Rearing temperature was significant for *ldhba* (ANOVA, *p* < 0.05, n = 48) and for *hsp70.1* ploidy, rearing temperature and the interaction were significant (ANOVA, *p* < 0.05, n = 47). Different letters indicate significant differences between groups (Tukey’s post-hoc test, (*p* < 0.05, n = 48).

Only the significance of *hsp70.1* changed using the combined index of *rps11* and *actb1* to normalize relative expression, compared to using all six housekeeping genes for normalization. Ploidy, rearing temperature and the interaction
